# Case report: Low-dose interleukin-2: a treatment of bullous pemphigoid with predominantly perifollicular blistering caused by PD-1/PD-L1 inhibitor

**DOI:** 10.3389/fimmu.2024.1496413

**Published:** 2024-12-10

**Authors:** Yingyue Zhang, Xianwei Cao, Jianbo Tong

**Affiliations:** ^1^ Department of Dermatology, The First Affiliated Hospital, Jiangxi Medical College, Nanchang University, Nanchang, China; ^2^ Institute of Dermatology, Jiangxi Academy of Clinical Medical Sciences, Nanchang, China

**Keywords:** bullous pemphigoid, Low-dose IL-2, PD-1/PD-L1 inhibitor, perifollicular blistering, immunotherapy

## Abstract

**Objectives:**

This study aimed to evaluate the efficacy of low-dose interleukin (IL-2) treatment for bullous pemphigoid (BP) caused by anti-programmed cell death protein 1/ligand 1 (PD-1/PD-L1) inhibitors.

**Methods:**

Low-dose IL-2 treatment was standardized for BP. The Bullous Pemphigoid Disease Area Index (BPDAI), 5D-Itch Scale (5D-IS), and Dermatology Life Quality Index (DLQI) were recorded before and after treatment, and hexachromatic lymphocytes, regulatory T cells (Treg cells), and cytokines were measured.

**Results:**

A significant decline in the BPDAI score, 5D-IS, and DLQI score was observed following treatment. The count of B-cells, CD4+ T-cells, CD8+ T-cells, Treg cells, and the levels of cytokines (IL-4, -8, and -10) were significantly downregulated in comparison to baseline measurements.

**Conclusion:**

Low-dose IL-2 could be an effective therapeutic choice for treating BP caused by PD-1/PD-L1 inhibitors.

## Introduction

Dermatologic immune-related adverse events are among the most common, affecting approximately 30% of patients receiving PD-1/PD-L1 inhibitors (1). Bullous pemphigoid (BP), although infrequently reported in this context, presents significant therapeutic challenges.

Interleukin (IL)-2, being a widely recognized pro-inflammatory cytokine, activates the immune system’s effector arm by stimulating a diverse spectrum of cells, including effector T (Teff) cells, memory cells, and natural killer cells (2). *In vitro*, sensitivity testing revealed the biomodulatory effect of IL-2 on Teff and Treg cells (3). High dosages increase Teff cells, promoting inflammation, while low doses boost Treg cells, exerting an anti-inflammatory effect. Some studies have confirmed the reduction of Treg cells in peripheral blood in patients with BP (4–6). The amplifying effect of low-dose IL-2 on Treg cells suggests its therapeutic potential in BP. Previous studies have suggested low-dose IL-2 as an effective treatment for several autoimmune diseases. However, the effect of low-dose IL-2 on BP caused by PD-1/PD-L1 inhibitors remains to be elucidated.

Herein, we report a case of a 31-year-old female patient with lung metastases from hepatocellular carcinoma who developed BP during immunotherapy with karelizumab (Erika). Following a standardized treatment approach, the patient was innovatively treated with low-dose IL-2 leading to a favorable response.

## Case report

**Figure 1:**
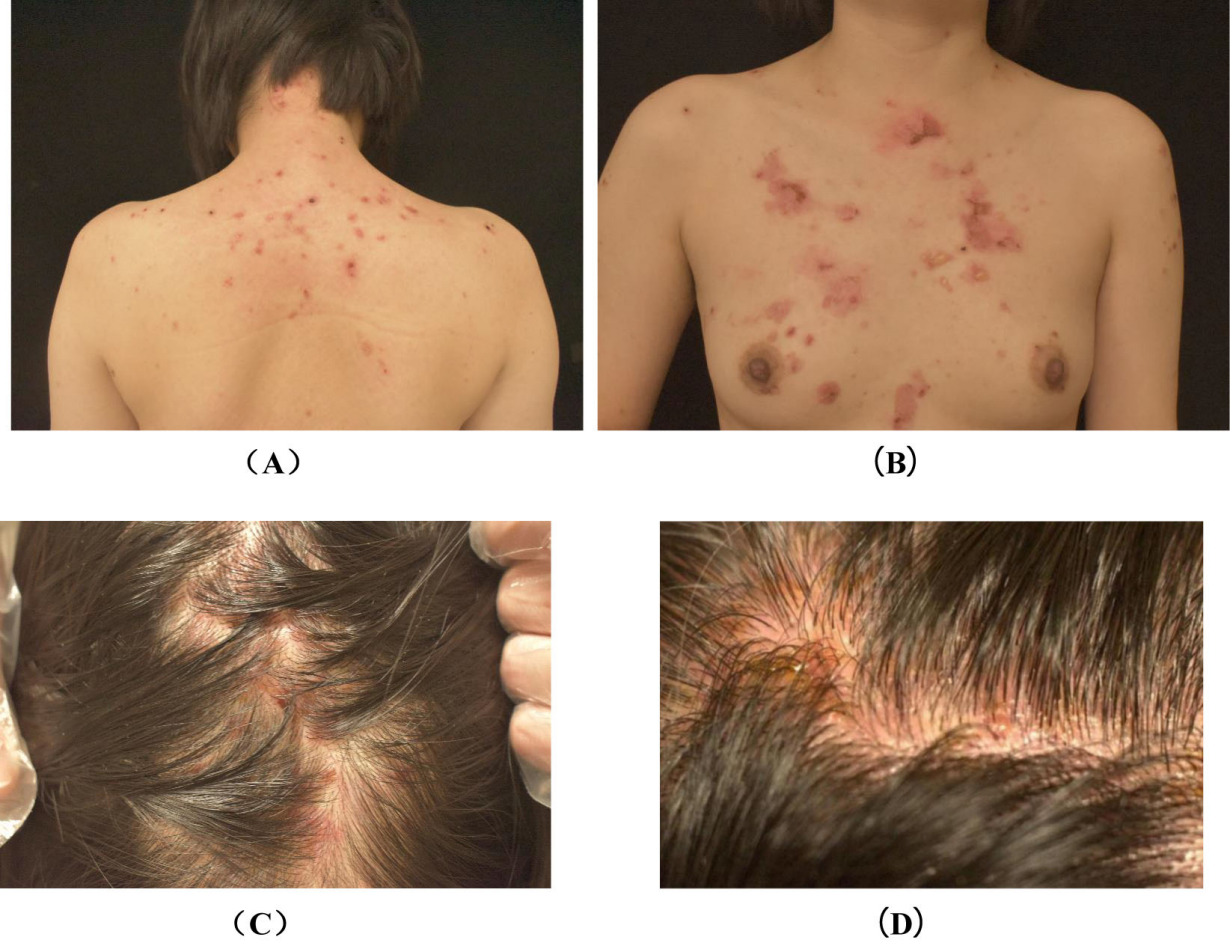
Photographs of back lesions **(A)**, chest lesions **(B)** and scalp lesions **(C, D)** before treatment. **(A)**: erythematous and papules on the back, as well as scabs that remain after the blisters have broken. **(B)**: erythematous, discrete tense blisters on the chest, followed by scabs that remain after the blisters have broken. **(C, D)**: diffuse erythema, fluid-filled blisters and scabs are seen on the scalp.

A 31-year-old female patient was diagnosed in January 2022 with hepatocellular carcinoma lung metastasis. The patient commenced with the PD-1/PD-L1 inhibitor karelizumab (Erika) in February 2022. She had no prior history of associated dermatologic or autoimmune diseases. Approximately 15 months later, erythematous, tense, transparent blisters began to appear on the body, primarily on the back, chest, and scalp, accompanied by significant itching (**Figure 1**). However, during this period, treatment was continued due to the low incidence of rashes and Erika’s positive effects. Meanwhile, the patient was administered oral doxycycline hydrochloride and topical hormone. In November 2023, the patient was hospitalized in our department due to the increasing number of new blisters per day and the expansion of rashes.

**Figure 2:**
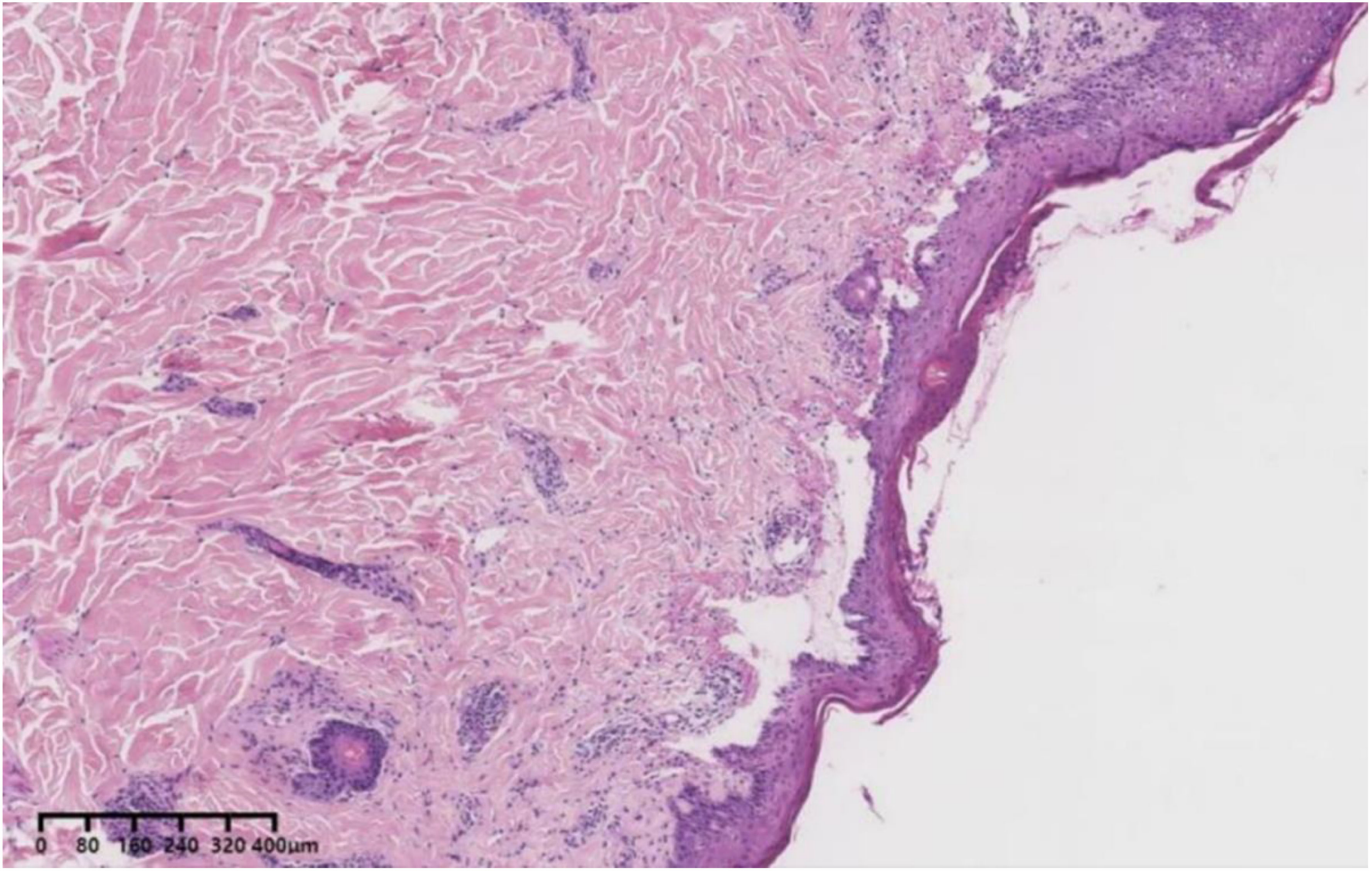
A 31-year-old female patient with blistering pemphigoid presenting as subepidermal blisters surrounded by eosinophilic infiltration (Hematoxylin and eosin).

To confirm the diagnosis, we performed an antibody test for BP and skin biopsy, which revealed elevated levels of the BP180 antibody IgG (> 200R U/mL) while the BP230 antibody IgG was negative. Direct skin immunofluorescence revealed IgG (+), IgM (-), C3 (+), and IgA (-). Moreover, the pathological examination indicated subepidermal blisters with eosinophilic infiltration (**Figure 2**), leading to BP diagnosis. Considering that tumor-induced paraneoplastic pemphigus (PNP) may also exhibit the aforementioned clinical features, we further refined the indirect immunofluorescence test using murine bladder epithelium as the substrate. Since autoantibodies that can recognize complex squamous epithelium, migrating columnar epithelium, and monolayer epithelial proteins are present in the sera of patients with PNP, serum from suspected patients is immediately twofold diluted, and indirect immunofluorescence is performed using murine bladder as a substrate. Immunofluorescence is usually found between the bladder epithelial acanthocytes. In this case, no fluorescence was detected between the spiny cells. Considering the patient’s history of immune checkpoint inhibitor use and the features of delayed skin lesions, we ultimately diagnosed PD-1/PD-L1 inhibitor-induced BP.

Considering the adverse effects associated with long-term hormone and immunosuppressant administration, a treatment plan was devised to address BP that involved subcutaneous administration of the standard course of low-dose IL-2 in combination with topical strong glucocorticoid and oral doxycycline hydrochloride. Specifically, IL-2 (0.8 MU) was administered subcutaneously every other day for seven times for one course of treatment, followed by the next courses administered at two-week intervals for three courses. The dose of topical halometasone was 10–20 g per day, while oral doxycycline hydrochloride was administered at a dose of 100 mg twice daily.

Despite the immune-related adverse event (BP), the patient responded well to karelizumab. According to established guidelines, BP resulting from immune checkpoint inhibitors is graded (7, 8): Grade I does not require discontinuation of immune checkpoint inhibitor therapy; grade II may require suspension of therapy, consultation with the dermatologist about treatment options, and assessment of the timing for resumption of immune checkpoint inhibitor therapy. This patient was diagnosed with Grade II lesions and treated with low-dose IL-2 during a temporary break in karelizumab therapy, resulting in rapid control of her symptoms. Based on the patient’s current manageable skin lesions, the decision to continue karelizumab was made in consultation with the oncologist.

**Figure 3:**
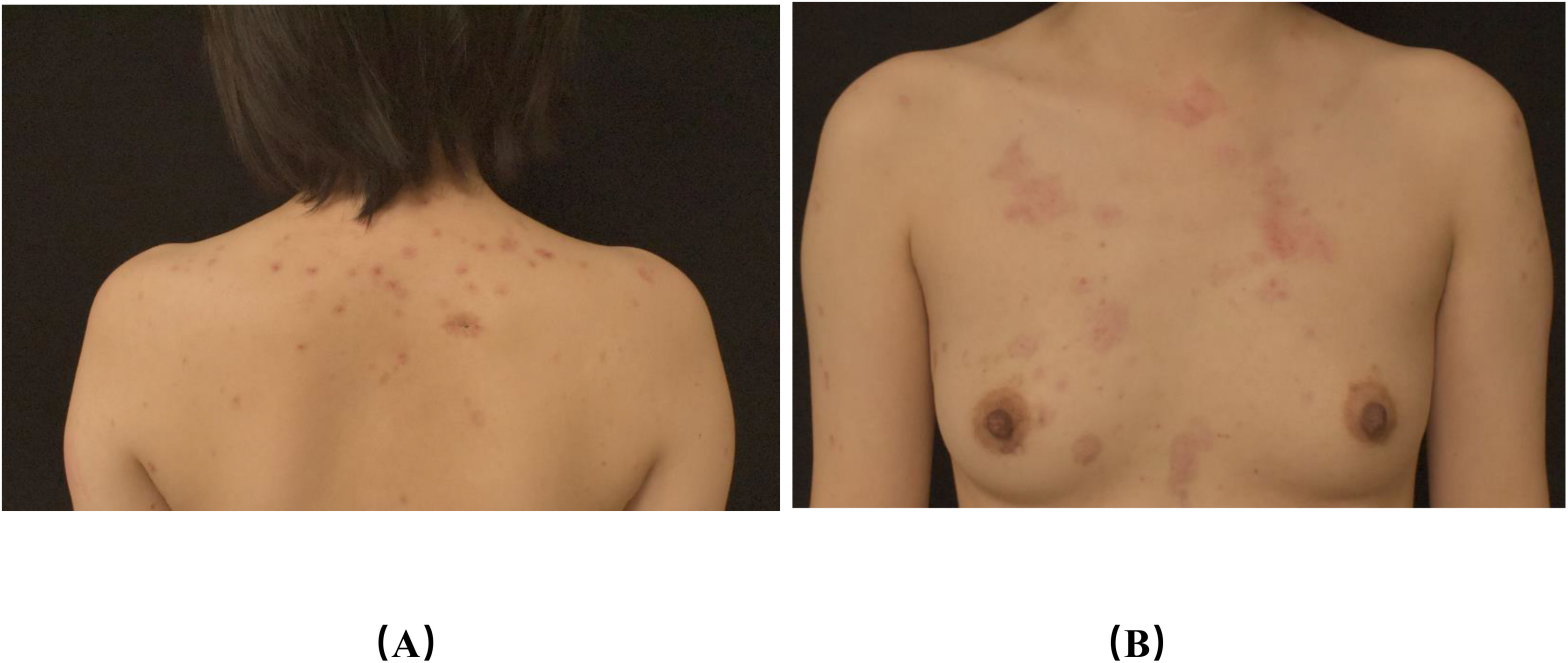
Photographs of back lesions **(A)**, chest lesions **(B)** after treatment **(A, B)**: after treatment, the erythema on the chest and back became milder than before, and all the scabs fell off.

To evaluate treatment efficacy, we assessed the Bullous Pemphigoid Disease Area Index (BPDAI) score, the 5D-Itch Scale (5D-IS), and the Dermatology Life Quality Index (DLQI) score before and after treatment and identified hexachromatic lymphocytes, Treg cells, and cytokines. Antibody levels were not measured after treatment; however, clinical observations were followed closely. Following the treatment with low-dose IL-2 alongside topical strong glucocorticoid and oral doxycycline hydrochloride, the blisters rapidly dried and crusted. Substantial healing was observed after two weeks, and the patient achieved disease control (**Figure 3**). The BPDAI score, 5D-IS, and DLQI score demonstrated a significant decline post-treatment. After treatment, there was a reduction in the absolute counts of B-cells, CD4+ T-cells, CD8+ T-cells, and Treg cells, with levels of IL-4 and -8 decreasing to normal, while IL-10 exhibited a slight decline (**Tables 1**–**4**). During the low-dose IL-2 treatment period, the patients were on schedule for karelizumab, demonstrating the effectiveness of a low-dose IL-2 regimen. We followed up for 10 weeks, after which the patient ceased treatment with IL-2, doxycycline hydrochloride, and topical potent hormones.

**Table 1:** Scores before and after treatment.

Time	BPDAI	5D-IS	DLQI
pre-treatment	39	33	15
post-treatment	9	16	2

BPDAI, the bullous pemphigoid disease area index; 5D-IS, 5D itch scale; DLQI, dermatology life quality index.

**Table 2:** Treg cells before and after treatment.

Items	Pre-treatment	Post-treatment	Frame of reference
Total T lymphocytes	63.60↓	54.45↓	64.19-75.77%
CD4+ T cells	37.25	29.93↓	30.09-40.41%
CD3+CD4+CD25+CD127dimTREG/	8.13	4.38	4-9%

↓, below frame of the reference.

**Table 3:** Relevant cytokines before and after treatment.

Items	Pre-treatment	Post-treatment	Frame of reference
Interleukin-4	4.31↑	2.14	0-4.19 pg/ml
Interleukin-8	115.23↑	4.21	0-15.71 pg/ml
Interleukin-10	3.63	2.02	0-4.50 pg/ml

↑, above frame of the reference.

**Table 4:** T-cells and B-cells before and after treatment.

Items Absolute count	Pre-treatment	Post-treatment	Frame of reference (units/UI)
CD4+ T cells	1143	443	331-1293
CD8+ T cells	653	216↓	228-941
B Cell	451	287	111-493

↓, below frame of the reference.

## Discussion

PD-1/PD-L1 inhibitor-associated BP is rare, with most available studies comprising case reports or small case series from a single institution (9). The BP pathogenesis related to PD-1/PD-L1 inhibitor therapy remains unclear, with two main ideas proposed. Some studies suggest that it is linked to the presence of common antigens at the junction of the cancer cells and the dermis of the skin (10). This mechanism is thought to involve the body’s hyperactive T lymphocytes, targeting BP180 on both tumor cells and the basement membrane. Additionally, the phenomenon of epitope expansion has been postulated (11). In contrast to conventional BP, BP caused by PD-1/PD-L1 inhibitors has a latency period averaging 6.25 months, with a range from 2 weeks to 20 months (12). In our case, the patient exhibited erythema and blister formation approximately 15 months after initiating karelizumab therapy, and these were most prevalent on the chest, back, and scalp—areas abundant in hair follicles—suggesting that the inflammation induced by the PD-1/PD-L1 inhibitor may have led to the exposure of hair follicle antigens, resulting in a loss of immune privilege (13).

From a therapeutic perspective, the clinical course of BP associated with PD-1/PD-L1 inhibitors differs from that of conventional drug-induced BP, which typically subsides abruptly following drug discontinuation. Conversely, BP associated with PD-1/PD-L1 inhibitors may persist for months after discontinuation due to ongoing immune activation by immune checkpoint monoclonal antibodies *in vivo*. Consequently, systemic glucocorticoid therapy is often required for moderate-to-severe BP caused by PD-1/PD-L1 inhibitors. However, long-term use of systemic glucocorticoid may lead to potentially fatal side effects; therefore, we opted for low-dose IL-2 therapy. Initially used in oncology, IL-2 has demonstrated significant benefits in patients with cancer (14). Recently, IL-2 has been increasingly used in treating autoimmune diseases, which may involve the following mechanisms: (i) Selective activation and expansion of Treg cells (15–20); (ii) inhibition of the differentiation of follicular helper T and helper T (Th17) cells that produce IL-17 (15, 20–22). Based on the safety of low-dose IL-2 in autoimmune diseases and its regulatory effects on T cells, we applied it over-the-counter to PD-1/PD-L1 inhibitor for treating BP and achieved good efficacy.

As the patient was treated with potent topical steroids and tetracycline, the definitive efficacy of IL-2 treatment for BP remains to be elucidated. Initially, the rapid progression of the disease persisted despite the application of potent topical steroids and tetracycline. However, after IL-2 treatment, the rash was controlled more rapidly, and the pruritus was significantly reduced. However, the exact mechanism of action remains unclear and requires further investigation for support.

## Conclusion

Low-dose IL-2 could be an effective therapeutic choice for treating BP caused by PD-1/PD-L1 inhibitors.

## Data Availability

The original contributions presented in the study are included in the article/supplementary material. Further inquiries can be directed to the corresponding author.
